# Design of High Temperature Complex Dielectric Properties Measuring System Based on XGBoost Algorithm

**DOI:** 10.3390/ma13061419

**Published:** 2020-03-20

**Authors:** Yuanyuan Wu, Li Wu, Huacheng Zhu, Tao Hong

**Affiliations:** 1 College of Information Science & Technology, Chengdu University of Technology, Chengdu 610000, China; wuyuanyuan@cdut.edu.cn; 2 College of Electronic and Information Engineering, Sichuan University, Chengdu 610065, China; wuli1307@scu.edu.cn (L.W.); hczhu@scu.edu.cn (H.Z.); 3 School of Electronic Information Engineering, China West Normal University, Nanchong 637002, China

**Keywords:** dielectric property, microwave interferometer, XGBoost algorithm

## Abstract

This paper aims to propose an online relative complex permittivity measurement system at high temperature based on microwave interferometer. A ridge waveguide with a TE_10_ mode was used in which the sample was heated and measured simultaneously at a frequency of 2450 MHz, and the microwave interferometer is used to collect the amplitude and phase difference of the incident signal. The Extreme Gradient Boosting (XGBoost) algorithm trained by the corresponding simulation data is used to construct the inversion model to calculate the complex dielectric coefficient of the tested material. Besides, this paper uses linear regression algorithm (LR) to calibrate the measurement system in order to improve the measurement accuracy. The entire system was tested using different materials at room temperature, and the maximum error of the measurement accuracy is less than 8% compared to the theoretical data. The robustness of the entire system was also tested by measuring Macor materials up to 800 °C. This proposed method provides an effective way to understand the mechanism between microwaves and matter at high temperatures.

## 1. Introduction

The dielectric property of material mainly relies on the frequency and temperature. In the high power application of microwaves, the microwave frequency is usually fixed, and the interaction between microwaves and matter at different temperature, especially high temperature, should be investigated [1,2,3,4,5]. Meanwhile, in the microwave heating process, thermal runaway always happens. Therefore, it is essential to study the complex permittivity of matter at high temperature in order to avoid the issue of thermal runaway and guide the design of the microwave equipment [6].

The measurement of relative permittivity of materials in high temperature environment has very important requirements in the fields of aerospace, metallurgy, and chemical industry [7,8,9,10,11,12]. The research on electromagnetic parameter measurement has also been the focus of attention in the fields of bioelectromagnetism and materials science [13,14,15,16]. For example, when the aircraft passes through the atmosphere, the aircraft will rub against the air, which will generate a large amount of heat, so that the surface temperature of the radome reaches about 2400 °C. Therefore, when designing the radome, it is necessary to measure the relative complex permittivity of the material of the radome in the high temperature range to ensure that the temperature does not affect the transmission of electromagnetic waves when the temperature rises. In addition, microwaves have also been widely used in the metallurgical industry [17,18,19,20]. The temperature of microwave metallurgy can achieve microwave high-temperature sintering, calcination decomposition, reduction, and synthesis at several thousand degrees Celsius. Therefore, in microwave metallurgy, the material needs to be in a high temperature state. Whether to absorb the microwave to make a judgment, so accurate measurement of the high temperature relative complex permittivity of the metallurgical material is very important for microwave metallurgy.

In the study of high-temperature dielectric measurement, JG Powles used a rectangular waveguide as a measuring device in the middle of the last century, connected the load at the waveguide end, and then placed the sample under test in the test waveguide, passing the electricity in the measurement system. The parameters complete the relative complex permittivity measurement of polycrystalline ceramic materials at 0.95 GHz and 2.4 GHz [21]; Vasundara V. Varadan designed a high-temperature relative complex permittivity measurement system based on free space method in their paper [22]. The system can measure the relative permittivity of materials with a frequency of 5.85–40 GHz and a temperature of 850 °C. The system uses the pair of point-focusing antennas to transmit and receive electromagnetic waves by heating the measured substances in a high-temperature furnace during measurement, and measures the parameters of the experimental system $\left|S_{21}\right|$ by a vector network analyzer, and then calculates the relative complex permittivity of the material. Yuma Katsuta designed a set of high temperature relative complex permittivity measurement system with a frequency of 2450 MHz using a cylindrical cavity in 2007 [23], by measuring the signal of the vector network analyzer. The power amplifier is used for amplification, and then the measured substance is heated by microwave heating, and the maximum heating power is up to 100 W. The relative complex permittivity of the measured substance is calculated by the scattering parameter of the cylindrical resonator. Arai et al. designed two different dielectric measurement systems to measure the high temperature relative complex permittivity of ceramic materials up to 1200 °C [7].

This paper proposes an on-line temperature relative complex permittivity measurement system at the frequency of 2450 MHz based on microwave interferometer. A ridge waveguide is designed as the core device of the system, where the tested materials can be placed. The scattering parameters are measured using the microwave power and microwave interferometer. The Extreme Gradient Boosting (XGBoost) [24] algorithm is used to predict the relative complex permittivity of the tested materials by performing regression training on data sets which are collected by finite difference time domain (FDTD) [25] simulation. Furthermore, the system uses linear regression algorithm (LR) [26] to calibrate the system to improve the measurement accuracy. The measurement system also integrates a data acquisition network to realize the automatic acquisition of measurement data for easy operation. The feasibility of the system was verified at room temperature and high temperature. Measurements on relative complex permittivity of the alcohols and Macor materials are also performed to evaluate the performance of the system. The measurement result proved the feasibility of the measurement system.

## 2. System Design

**Figure 1:**
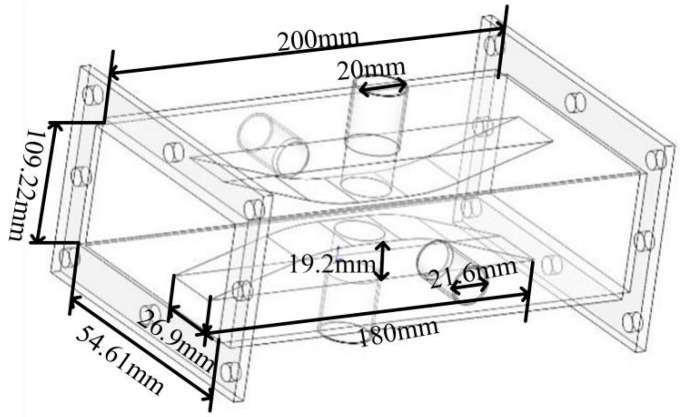
Schematic of ridge waveguide.

In the system, the ridge waveguide is used for heating and measuring, and the structure of ridge waveguide is shown in Figure 1. The ridge waveguide has a cut-off waveguide on the upper and lower sides, where the test tube is placed. The cut-off waveguide can pass electromagnetic waves above the cutoff frequency, filter out electromagnetic waves below the cutoff frequency to achieve electromagnetic shielding [27]. Combined with the FDTD (finite difference time domain) simulation, the size of the ridge waveguide is constantly optimized, and the final size of the designed ridged waveguide is shown in Table 1.

**Figure 2:**
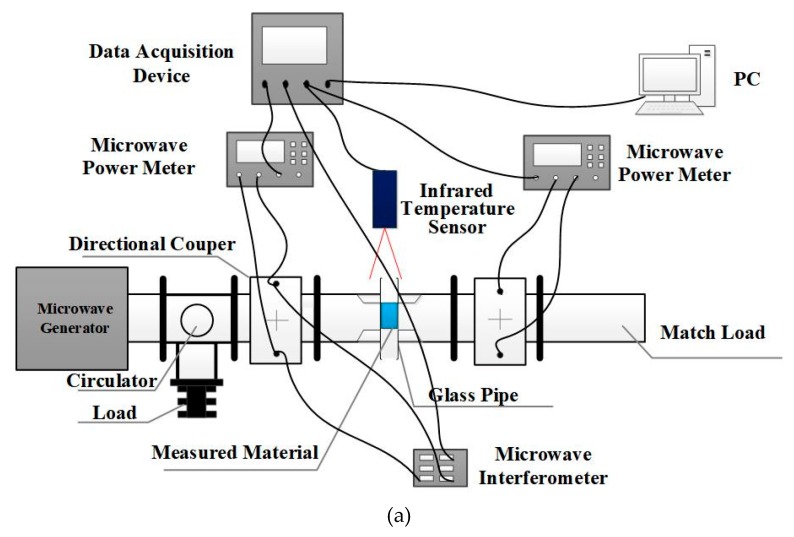

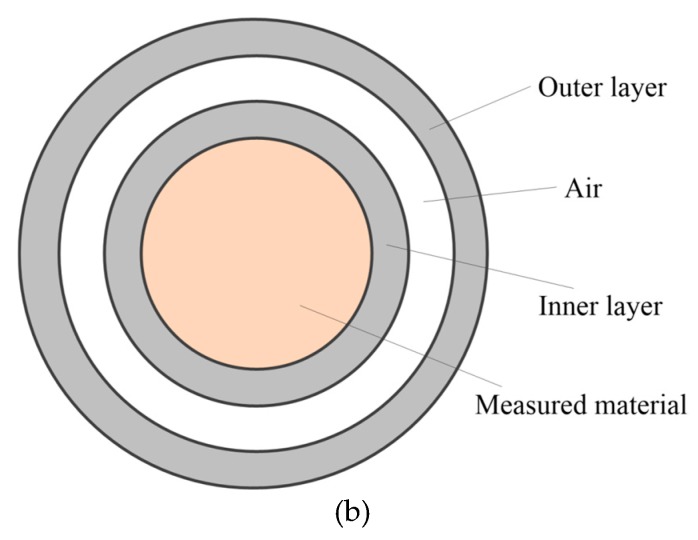
Measurement system diagram: (**a**) the whole system, (**b**) top view of the glass tube.

The system consists of a solid-state source, a circulator, a dual directional coupler, a ridge waveguide, a matched load power meter, an infrared thermometer, an interferometer module, and a data acquisition module. The measurement system is shown in Figure 2a. The material to be tested is placed in a quartz glass tube located at the center of the ridge waveguide. In order to solve the problem of non-uniform heating, a double-layer glass tube is used as shown in Figure 2b. The thickness of the inner and outer layer is 1.5 mm, the thickness of the air layer is 2 mm, and the diameter of the measured material is 10 mm. The power generated by the microwave source is coupled to both ends of the ridge waveguide via a circulator and a dual directional coupler, respectively. The power meter is also connected to a dual directional coupler to measure the input power, output power, and transmission power of the ridge waveguide. Based on the measured power, $\left|S_{11}\right|$ and $\left|S_{21}\right|$ at both ends of the ridge waveguide in the current state can be calculated. By adding a microwave interferometer module to the measurement system, the real-time measurement of the phase of $S_{11}$ can be accomplished while using microwave heating.

**Figure 3:**
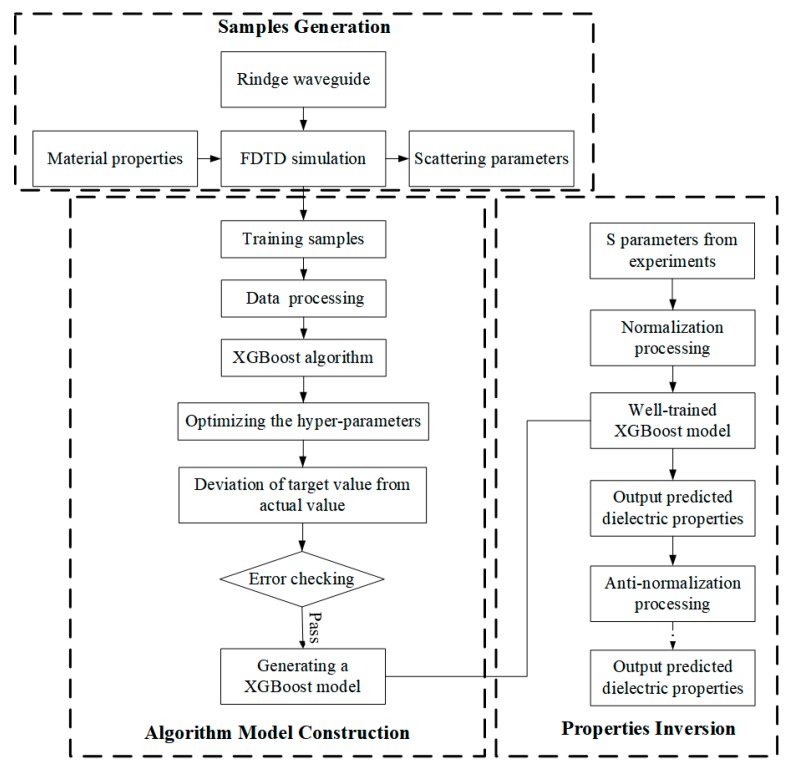
Inversion flow chart.

During the heating process, the relative complex permittivity of the material changes with the increase of temperature, and the absorption and reflection of the microwave also change. The change of the reflected power and the transmitted power means the change of $\left|S_{11}\right|$, $\left|S_{21}\right|$, and $\varphi_{S_{11}}$, indicating that the relative complex permittivity of the material have a certain correspondence with the *S* parameters. Therefore, the XGBoost algorithm is used to simulate the corresponding relationship. The process of predicting relative complex permittivity with XGBoost algorithm is shown in Figure 3. First, the data sets with labels used for algorithm training are generated by FDTD simulation, then XGBoost machine learning model is established by Python language, automatic parameter optimization is carried out by grid search method [28]. Finally, the *S* parameters measured by experiments can be sent to the well-trained XGBoost algorithm model, then the relative complex permittivity can be predicted.

## 3. Methodology

### 3.1. Samples Generation

**Figure 4:**
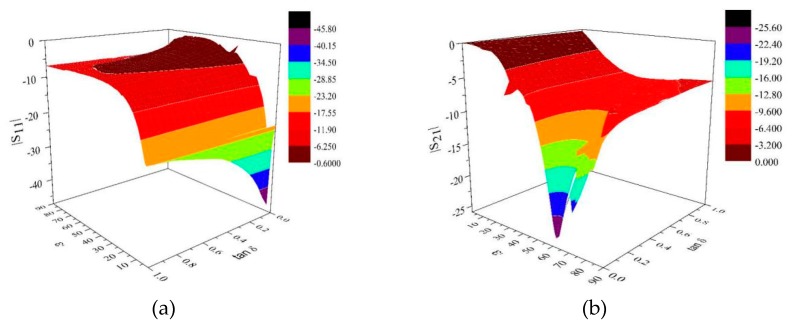
(**a**) Relationship between $\left|S_{11}\right|$ and relative complex permittivity; (**b**) Relationship between $\left|S_{21}\right|$ and relative complex permittivity.

In the measurement of high temperature relative complex permittivity, the construction of sample space is particularly important. When XGBoost algorithm is used to inverse the dielectric properties, it often encounters multi-value problems, which means that different relative complex dielectric coefficients correspond to the same *S* parameters. In order to solve this problem, the system optimizes the structure of the ridge waveguide model during design, so that the relative complex dielectric coefficient corresponds to the *S* parameter one by one. The monotonicity of the data is ensured by continuously optimizing the height and width of the ridge and the diameter of the quartz tube combined with the FDTD algorithm. Figure 4a,b shows the relationship between $\left|S_{11}\right|$, $\left|S_{21}\right|$ and dielectric properties of the final ridged waveguide, respectively.

It can be seen from Figure 4 that when the real part of the dielectric is in the range of 1–5, there is a multivalued phenomenon of $\left|S_{11}\right|$, but the monotonicity is better when the range of the real part of the dielectric is 5–40. However, $\left|S_{21}\right|$ is monotonous in the range of 1–40, basically there is no multi-value phenomenon.

**Figure 5:**
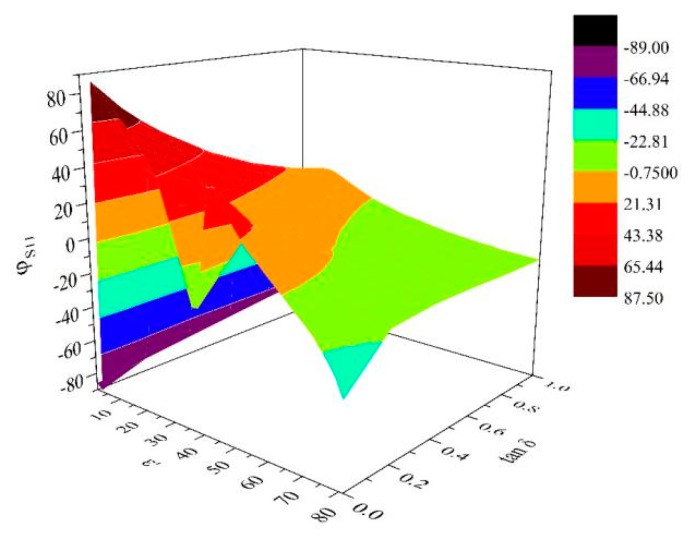
Relationship diagram of $\varphi_{S_{11}}$ and relative complex permittivity.

In order to reduce the multi-value phenomenon of the inversion calculation better, the XGBoost adopts three input and two output network structures, with $\left|S_{11}\right|$, $\left|S_{21}\right|$ and $\varphi_{S_{11}}$ as the input vectors, and the real and imaginary parts of the relative complex permittivity as the output vectors. So there is a certain requirement for the monotonicity of $\varphi_{S_{11}}$. Therefore, the relationship between $\varphi_{S_{11}}$ and the relative complex permittivity in the ridge waveguide is calculated with FDTD simulation, as shown in Figure 5.

It can be seen from the figure that with the real part of the relative complex permittivity, $\varphi_{S_{11}}$ decreases gradually. When the real part of the relative complex permittivity is less than 30, there is a multi-valued phenomenon of $\varphi_{S_{11}}$. However, the combination of $\left|S_{11}\right|$ and $\left|S_{21}\right|$ can effectively avoid the influence of single parameter and multi-value on algorithm inversion, that ensure the accuracy of calculation results.

### 3.2. Algorithm Model Construction

This paper used the XGBoost algorithm as the core algorithm for inverting relative complex permittivity. Boosting is a machine learning technique algorithm. It is one of the Boosting algorithms, a boost library with linear scale solver and tree learning algorithm developed by Tianqi Chen of the university of Washington in 2016 [24]. Its biggest characteristic is that it can call CPU manually for multithreading parallel computing, so it is more than ten times faster than the same algorithm under the same condition [29]. The algorithm is also improved to achieve higher accuracy. The steps can be described as follows:

1. Add a regularization term to the target function:

(1) $$Loss\left(x_{i}\right)=\sum_{i=1}^{n}l\left({\hat{y}}_{i},y_{i}\right)+\sum_{i=1}^{t}\Omega \left(f_{i}\right)$$

where

(2) $$\Omega \left(f\right)=\gamma T+\frac{1}{2}\lambda \sum_{j=1}^{T}\omega_{j}^{2}$$

(3) $$f_{t}\left(x\right)=w_{q\left(x\right)}$$

Loss is the error function, representing the fitted data of the model. Ω is a regularization term, which represents the parameters of the penalty complex model and is used to solve over fitting. In this paper, $y_{i}$ and ${\hat{y}}_{i}$ represent the actual and predicted relative complex permittivity ($\varepsilon_{r}^{\prime }$,$\varepsilon_{r}^{\prime \prime }$) respectively. x represents scattering parameters ($\left|S_{11}\right|$,$\left|S_{21}\right|$,$\varphi_{S_{11}}$).

2. Second-order Taylor expansion is used to expand the error function at ${\hat{y}}_{i}^{t-1}$:

(4) $$loss\approx \sum_{i=1}^{n}l\left(y_{i},{\hat{y}}_{i}{}^{t-1}\right)+g_{i}f_{t}\left(x_{i}\right)+\frac{1}{2}h_{i}f_{t}{\left(x_{i}\right)}^{2}]+\sum_{i=1}^{t-1}\Omega \left(f_{i}\right)+\Omega \left(f_{t}\right)$$

where

(5) $$g_{i}=\partial_{{\hat{y}}^{\left(t-1\right)}}l\left(y_{i},{\hat{y}}_{i}{}^{t-1}\right)$$

(6) $$h_{i}=\partial^{2}{}_{{\hat{y}}^{t-1}}L\left(y_{i},{\hat{y}}_{i}{}^{t-1}\right)$$

3. Removing all constant terms and substituting Equation (3) and regular terms (2) into Equation (4), we can get:

(7) $$loss\approx \sum_{i=1}^{n}\left[g_{i}w_{q\left(x_{i}\right)}+\frac{1}{2}h_{i}w_{q\left(x_{i}\right)}^{2}\right]+\gamma T+\lambda \frac{1}{2}w_{j}^{2}$$

4. Defining the sample set on the *j*-th leaf node as: $I_{j}=\left\{i|q\left(x_{i}\right)=j\right\}$, then converting the sample accumulation operation to operation on the leaf node:

(8) $$loss\approx \sum_{j=1}^{T}\left[\left(\sum_{i\in I_{j}}g_{i}\right)w_{j}+\frac{1}{2}\left(\sum_{i\in I_{j}}h_{i}+\lambda \right)w_{j}^{2}\right]\gamma T$$

This paper builds the XGBoost machine learning model based on the Python programming language. The mean squared error (*MSE*), the mean absolute error (*MAE*), and the coefficient of determination ($R^{2}$) in the metrics module in s-klearn library are called to evaluate the prediction error [30]. The *MSE* and *MAE* estimated over $n_{samples}$ is defined as:

(9) $$MSE(y,\hat{y})=\frac{1}{n_{samples}}\sum_{i=0}^{n_{samples}-1}{\left(y_{i}-{\hat{y}}_{i}\right)}^{2}$$

(10) $$MAE(y,\hat{y})=\frac{1}{n_{samples}}\sum_{i=0}^{n_{samples}-1}\left|y_{i}-{\hat{y}}_{i}\right|$$

where ${\hat{y}}_{i}$ is the predicted value of the *i*-th sample, and $y_{i}$ is the corresponding actual value. The $R^{2}$ estimated over $n_{samples}$ is defined:

(11) $$R^{2}\left(y,\hat{y}\right)=1-\frac{\sum_{i=0}^{n_{samples}-1}{\left(y_{i}-{\hat{y}}_{i}\right)}^{2}}{\sum_{i=0}^{n_{samples}-1}{\left(y_{i}-\bar{y}\right)}^{2}}$$

where $\bar{y}=\frac{1}{n_{samples}}\sum_{i=0}^{n_{samples}-1}y_{i}$. $R^{2}$ provides an index of the degree of accuracy in the predicted samples. The best score of $R^{2}$ is 1. Besides, the smaller the *MSE* and *MAE*, the better the prediction.

After training the XGBoost model with the training data set obtained from FDTD simulation, the parameters of XGBoost model are determined by optimizing the values of *MSE*, *MAE,* and R², as shown in Table 2.

**Figure 6:**
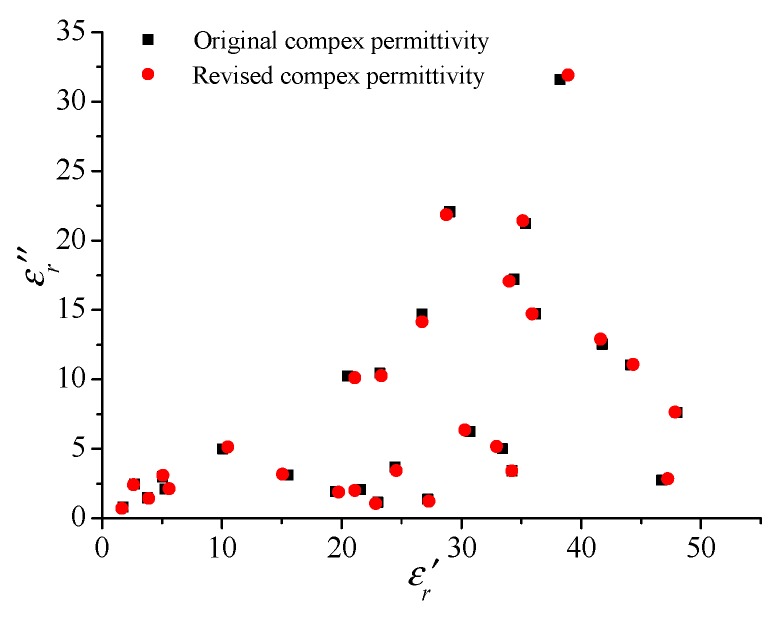
Comparison of the actual and reversed complex permittivity.

After the parameters of XGBoost model are obtained, different data from the training data set is selected to test this model, so that the prediction effect of the algorithm can be evaluated. Figure 6 shows the prediction of the real and imaginary parts of the relative complex permittivity. It can be seen from the above figure that the prediction error of the real part of the complex permittivity can be as low as 1.1%, and when the real part is less than 20, the predicted value is very accurate, the imaginary error is generally below 3%.

### 3.3. System Calibration

In order to ensure the accuracy of system measurement, the system needs to be calibrated before measurement to eliminate the error caused by system deviation. This paper uses linear regression algorithm in machine learning to calibrate the system [31]. The data of several groups of common and easily accessible chemical reagents are measured using the system without calibration. The results show that the measurement results of methanol and ethanol (reagent grade liquid, Chengdu Kelong Chemical Reagent Factory, Chengdu, China) are more accurate and stable, as shown in Table 3. Therefore, methanol, ethanol, and air test are combined with linear regression algorithm to calibrate the system. The calibration principle is as follows:

(12) $$\hat{S}(\omega ,S)=\omega_{0}+\omega_{1}S_{\mathrm{methanol}}+\omega_{2}S_{\mathrm{ethanol}}+\omega_{3}S_{\mathrm{air}}$$

Here, we define $\omega =\left\{\omega_{0},\omega_{1},\omega_{2},\omega_{3}\right\}$ as the parameter vector of system drift. $S=\left\{\left|S_{11}\right|,\left|S_{11}\right|,\varphi_{S_{11}}\right\}$ is the *S* parameters after system calibration. $S_{\mathrm{methanol}}$, $S_{\mathrm{ethanol}}$ and $S_{\mathrm{air}}$ represent labeled data sets of methanol, ethanol, and air respectively, that are the *S* parameters measured and simulated by FDTD algorithm.

The drift parameter vector ω of the system can be determined by algorithm and this data. Therefore, when the calibrated system is used for measurement, the calibrated *S* parameters can be obtained by inputting the measured *S* parameters, and then sending them to the well-trained XGBoost model for inversion. The results after calibration with regression algorithm are also shown in Table 3.

**Figure 7:**
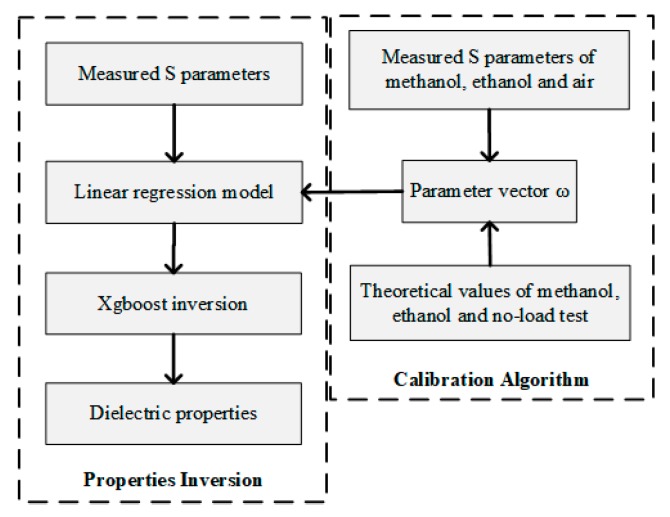
The schematic diagram of system calibration.

The results show that the system error reduced after calibration. Therefore, in order to improve the accuracy of system measurement, it is necessary to measure the *S* parameter values of methanol, ethanol, and air before experiment to calibrate the system. The schematic diagram of system calibration with linear regression algorithm is shown in Figure 7.

## 4. Measured Results and Discussion

Measurements are conducted to verify the system reliability under room temperature and dynamic high temperatures respectively.

### 4.1. Room Temperature Measurement

The easy-to-access chemical substances in the laboratory are used for the experiment. As shown in Table 4, the relative complex permittivity of methanol and ethanol and the relative complex permittivity of mixed solutions of different chemical solvents were tested using the system. The reference values are obtained by other experiments at the frequency of 2450 MHz [32], and the values of mixtures can be obtained by the Bruggeman formula [33]. The measurement errors are all within 8%, which basically meets the general measurement accuracy requirements.

### 4.2. High Temperature Measurement

**Figure 8:**
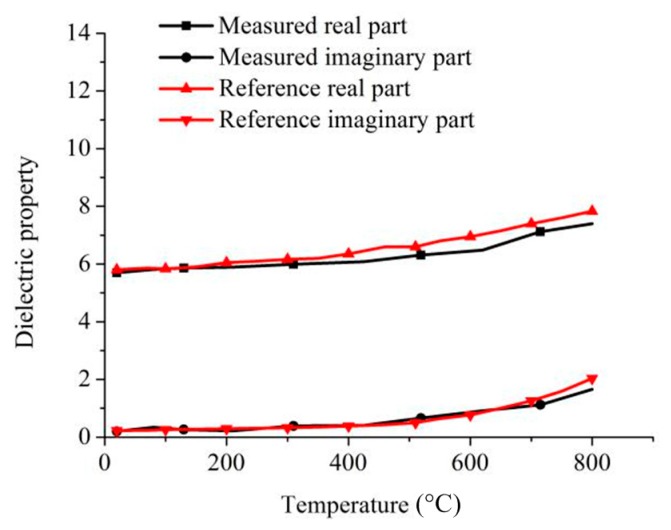
Comparison of measured and reference relative complex permittivity of Macor.

Macor materials that are less susceptible to deformation at elevated temperatures are used to verify the relative complex permittivity of the system at elevated temperatures. The experimental results were compared with the results in the reference [34]. The specific measurement results are shown in Figure 8.

It can be seen from the measurement results that when the temperature is below 400 °C, the measured values of the real and imaginary parts of the relative complex permittivity of Macor are close to those of the reference. When the temperature reaches 500 °C, the real and imaginary parts of the relative complex permittivity of Macor show a certain degree of increase. The measured real part has a certain deviation from the reference value, but the error is small. The value of the imaginary part of the electrical coefficient is still relatively close. When the temperature rises to 600 °C, the change trend of the imaginary part of the complex dielectric constant is still consistent with the reference. The measured value of the real part of the complex dielectric coefficient still has a certain deviation from the reference value, but the change trend is consistent. To some extent, the accuracy of the measurement of the system is proved, and compared with the measurement results in the previous chapter, after adding the phase measurement to the system, the relative complex permittivity of the Macor and the reference are calculated by inversion. The values are closer, indicating that the measurement accuracy of the system is improved compared to the measurement system of the previous chapter, and the measurement accuracy of the system is further optimized.

## 5. Conclusions

This paper designs an on-line high temperature relative complex permittivity measurement system at a working frequency of 2450 MHz. The core device of the system is a ridge waveguide, and the heating and relative complex permittivity of the sample to be tested are measured by the ridge waveguide; the scattering parameters in the experimental system are measured using a microwave power meter and a microwave interferometer, respectively, and the XGBoost model training is completed to construct an inversion calculation network to realize the calculation and measurement of the relative complex permittivity of the measured substance. The accuracy of the system measured at room temperature was verified using chemical solvents and their mixed solvents with known relative complex dielectric coefficients. The relative complex permittivity of Macor materials up to 800 °C was measured and compared, and the online high temperature relative complex was compared. The measurement results of the electrical coefficient measurement system and the data in the reference documents verify the measurement accuracy of the high temperature relative complex permittivity measurement system in the high temperature range.

## Figures and Tables

**Table 1:** Dimensions of the ridged waveguide.

Name	Ridge Width	Ridge Length	Ridge Height	Observation Hole Radius	Waveguide Length	Waveguide Diameter	Material Hole Radius
Size (mm)	26.9	180	19.2	10.8	200	54.61 × 109.22	10

**Table 2:** Parameters of Extreme Gradient Boosting (XGBoost) model.

Max_depth	Learning_rate	Objective	N_estimator	Booster	Gamma	Lambda
6	0.1	logistic	1000	gbtree	0.1	3
Subsample	$\varepsilon_{r}^{\prime }$ *MSE*	$\varepsilon_{r}^{\prime }$ *MAE*	$\varepsilon_{r}^{\prime }R^{2}$	$\varepsilon_{r}^{\prime \prime }$ *MSE*	$\varepsilon_{r}^{\prime \prime }$ *MAE*	$\varepsilon_{r}^{\prime \prime }R^{2}$
0.7	0.1683	0.2907	0.9982	0.00101	0.0244	0.9889

**Table 3:** Measurement results of methanol and ethanol.

Material	Property	Before Calibration	Ref [32]	Errors	After Calibration	Errors
methanol	$\varepsilon_{r}^{\prime }$	25.2	24.97	0.92%	25.11	0.56%
	$\varepsilon_{r}^{\prime \prime }$	14.26	14.48	1.51%	14.32	1.10%
ethanol	$\varepsilon_{r}^{\prime }$	8.28	8.94	7.38%	8.72	2.46%
	$\varepsilon_{r}^{\prime \prime }$	7.29	7.60	4.08%	7.45	1.97%

**Table 4:** Measurement results at room temperature. (“4 methanol + 1 ethanol” means the volume rate between methanol and ethanol is 4 to 1.)

Media	Measured $\varepsilon_{r}^{\prime }$	Reference $\varepsilon_{r}^{\prime }$	Errors	Measured $\varepsilon_{r}^{\prime \prime }$	Reference $\varepsilon_{r}^{\prime \prime }$	Errors
Ethanol	8.72	8.94	2.46%	7.45	7.60	1.97%
Methanol	25.11	24.97	0.56%	14.32	14.48	1.10%
4 methanol + 1 ethanol	20.75	21.03	1.33%	13.28	13.04	1.84%
2 methanol + 3 ethanol	13.25	14.09	5.96%	10.73	10.14	5.82%
4 methanol + 1 N-butanol	19.21	19.17	0.21%	11.66	11.12	4.86%
2 methanol + 3 N-butanol	9.31	9.13	1.97%	5.77	5.39	7.05%
